# Children's Self-Regulation and School Achievement in Cultural Contexts: The Role of Maternal Restrictive Control

**DOI:** 10.3389/fpsyg.2016.00722

**Published:** 2016-05-31

**Authors:** Mirjam Weis, Gisela Trommsdorff, Lorena Muñoz

**Affiliations:** ^1^Department of Psychology, University of KonstanzKonstanz, Germany; ^2^Department of Psychology, Universidad de ChileSantiago de Chile, Chile

**Keywords:** self-regulation, school achievement, culture, parenting, restrictive control

## Abstract

Self-regulation can be developed through parent-child interactions and has been related to developmental outcomes, e.g., such as educational achievement. This study examined cross-cultural differences and similarities in maternal restrictive control, self-regulation (i.e., behavior and emotion regulation) and school achievement and relations among these variables in Germany and Chile. Seventy-six German and 167 Chilean fourth graders, their mothers, and their teachers participated. Mothers and teachers rated children's behavior regulation with a subscale of the Strengths and Difficulties Questionnaire. Children reported their use of emotion regulation strategies on the Questionnaire for the Measurement of Stress and Coping. Mothers rated maternal restrictive control by answering the Parenting Practice Questionnaire. School achievement was assessed by grades for language and mathematics. Results showed higher behavior regulation of German children in comparison to Chilean children and a higher preference of restrictive parental control in Chilean mothers than in German mothers. Regression analyses revealed positive relations between children's behavior regulation and school achievement in Germany and in Chile. Further, in both cultural contexts, maternal restrictive control was related negatively to behavior regulation and positively to anger-oriented emotion regulation. In sum, the study showed the central function of behavior regulation for school achievement underlining negative relations of maternal restrictive control with children's self-regulation and school achievement in diverse cultural contexts. Culturally adapted interventions related to parenting practices to promote children's behavior regulation may assist in also promoting children's school achievement.

## Introduction

Self-regulation has become one of the most important and most frequently studied constructs in the whole field of psychology (Duckworth, 2011; Vohs and Baumeister, 2011). A wide range of studies has discussed the important function of self-regulation for positive developmental outcomes (Tangney et al., 2004; e.g., Moffitt et al., 2011). School achievement is one of the main phenomena that have been related to self-regulation. In spite of numerous studies on self-regulation, the role of cultural contexts on the development of self-regulation has been largely ignored (Trommsdorff and Cole, 2011; Trommsdorff, 2012), with most of the studies having been conducted in Northern American or European contexts. Specifically, relations between self-regulation and school achievement have rarely been studied in Latin American contexts. While Northern American and European countries can be described as independent cultural contexts, characterized by a high motivation for individual autonomy which is linked to self-regulation (Trommsdorff, 2009), the role of self-regulation in Latin American countries, is less clear. As Latin American countries are rapidly changing, independent and interdependent values may combine (e.g., Trommsdorff and Kornadt, 2003). Thus, it is important to study self-regulation in countries of socio-cultural change. Furthermore, insights into socialization conditions for children's development of self-regulation and adaptation to the school context by taking into account diverse cultural contexts are still lacking (Weis, 2015).

Parental responsiveness, autonomy support, and parental control have been investigated as parenting aspects related to the development of self-regulation (Grolnick and Ryan, 1989; Karreman et al., 2006). We decided to focus on maternal restrictive control since this parenting aspect might have a crucial negative impact on children's development of self-regulation (Kopp, 1982; Barber, 1996). Moreover, previous studies mostly investigated behavior regulation (or self-control) but largely neglected a wider conceptualization of self-regulation including behavior and emotion regulation. The present study aims to contribute to fill these gaps by investigating relations between maternal restrictive control, different aspects of self-regulation (i.e., behavior regulation, emotion regulation), and school achievement in a typical independent context (Germany) and in a context of cultural change (Chile). The introduction starts with the article's main focus on the importance of self-regulation for school achievement; then we discuss the role of maternal restrictive control for self-regulation and school achievement.

### Self-regulation and school achievement

We understand self-regulation as a skill and motivation for goal-directed behavior necessary to achieve individual needs in academic and social situations (Kopp, 1982; Karoly, 1993; Trommsdorff, 2009). To capture this complex construct adequately, we include behavior and emotion regulation in our research. Behavior regulation means to pay attention, follow rules, resist temptation, and inhibit impulsive behavioral reactions to comply with environmental demands (e.g., Calkins, 2007; McClelland et al., 2007). In spite of relatively stable differences between individuals in behavior regulation (Raffaelli et al., 2005), there is situation specific variance in behavior regulation within individuals depending on the context and the goals of individuals (Tsukayama et al., 2012). Emotion regulation, describes the processes which initiate, inhibit, avoid, maintain, or modulate emotions in order to achieve individual goals (Eisenberg and Spinrad, 2004). Here, we focused on emotion regulation strategies for coping with negative emotions. Lazarus and Folkman's transactional model of stress and coping distinguishes between emotion-oriented and problem-oriented strategies in coping with negative emotions (e.g., Lazarus and Folkman, 1984). While emotion-oriented strategies aim to reduce the negative emotional experience directly (e.g., relieving tension), problem-oriented strategies aim to change the situation which elicited the negative emotions (Lohaus et al., 2006). Thus, problem-oriented strategies include instrumental actions to solve the problem actively. In the present study, we included problem- as well as emotion-oriented strategies. Regarding emotion-oriented strategies, we focused on anger-oriented strategies which are instrumental to relieve the tension of anger, an “intense adaptive approach emotion that requires the mastery of efficient regulatory strategies for proper functioning” (Feldman et al., 2011, p. 310). Furthermore, anger regulation has been shown to differ among cultural contexts depending on the respective cultural values (Cole et al., 2006; Trommsdorff and Cole, 2011).

Self-regulation is conceived of as an important skill and motivation helping children to be successful in school (Blair, 2002). Past research has shown a positive relation of self-regulation with academic achievement (e.g., Calkins, 2007; McClelland et al., 2007). However, a more nuanced conceptualization of self-regulation, including its interrelated but separate aspects of behavior and emotion regulation (e.g., Raffaelli et al., 2005), has been largely ignored in previous studies.

Behavior regulation is necessary to remember and follow instructions and to concentrate on tasks without getting distracted. Thus, behavior regulation is essential to be successful in school (McClelland et al., 2007). Past studies in European and North American countries focusing on diverse age groups (preschoolers to high school students) already showed positive relations between behavior regulation and school achievement (e.g., McClelland et al., 2007; Weis et al., 2013). Furthermore, behavior regulation even accounts for variance in school achievement beyond the variance that is explained by intelligence (Duckworth and Seligman, 2005; Suchodoletz et al., 2009).

Besides behavior regulation, children have to regulate their emotions to engage in school-related activities. Adequate emotion regulation in the classroom improves several cognitive processes (e.g., memory, attention, planning, problem solving), which are essential for scholastic learning (Blair, 2002). Several studies in European and North American countries showed positive links between effective emotion regulation and school achievement in preschoolers (e.g., Graziano et al., 2007). As adaptive emotion regulation means to adopt situation specific strategies, problem- as well as emotion-oriented strategies may be effective depending on the situation (Lohaus et al., 2006). However, in the school context, a study with fourth to sixth graders (Mantzicopoulos, 1990) showed that problem-oriented strategies are more effective for school achievement than emotion-oriented strategies. Relations between anger-oriented strategies and school achievement have rarely been investigated in previous studies. In the present study, we focused on relations between anger-oriented strategies, problem-oriented strategies, and school achievement.

### Restrictive control and self-regulation

Self-regulation with its components behavior and emotion regulation develops from external to internal regulation (Kopp, 1982). Infants' behavior and emotions are regulated mostly by parents (external regulation). With increasing age, children acquire a set of regulation strategies which allows them to regulate their emotions and behavior in the absence of their caregivers (internal). Hence, it is evident that parenting plays a crucial role for the development of self-regulation. Previous studies have shown several relevant parenting aspects for the development of self-regulation, e.g., parental warmth, responsiveness, autonomy support, and parental control (Grolnick and Ryan, 1989; Davidov and Grusec, 2006; Karreman et al., 2006; Suchodoletz et al., 2011). Referring to Kopp's (1982) theory on the development of self-regulation, parental control with its aspects positive and “negative” control plays an important role. In the present study, we focused on “negative” control, labeled here as “restrictive” control. Restrictive control is defined as aggressive, strict, and critical parenting behavior, typically including anger, harshness, and intrusive control (Karreman et al., 2006). While positive control (i.e., guiding the child's behavior by limit-setting, instructing, and encouraging) may foster the development of self-regulation, restrictive control may undermine the child's internalization of autonomous regulation processes and therefore could negatively influence the development of self-regulation (Grolnick and Ryan, 1989; Karreman et al., 2006). In the present study, we have focused on restrictive control which has been shown in socialization research to be predictive of less autonomy and more internalizing problems in children (Barber, 1996). Previous studies also revealed that maternal restrictive control is negatively related to children's behavior regulation (see Karreman et al., 2006) and positively to anger-oriented emotion regulation (Feldman et al., 2011).

### Restrictive control, self-regulation, and school achievement

Further, maternal restrictive control has been shown to be associated negatively with school achievement (Dornbusch et al., 1987; Grolnick and Ryan, 1989). There is evidence that maternal restrictive control negatively influences both self-regulation and school achievement. Wong (2008) showed in a study with US-American adolescents that behavior regulation can mediate the link between parenting and school achievement. Therefore, we investigated whether the relation between maternal restrictive control and school achievement is mediated by both behavior and emotion regulation as aspects of self-regulation. Moreover, we extended the mediation models by controlling for intelligence, age, and gender. Further, we tested these mediation models in samples of German and Chilean fourth graders, to gain insights about the conditions and outcomes of self-regulation in cultural contexts.

### Restrictive control, self-regulation, and school achievement in cultural contexts

According to Trommsdorff (2009) cultural model of agency, self-regulation develops successfully when conforming to dominant cultural values and to cultural specific meanings of autonomy (personal and relational). Thus, self-regulation processes might differ cross-culturally due to cultural specific models of agency. Whereas, the independent model of agency implies self-regulation behavior based on its underlying motivation for individual autonomy (e.g., achieve own goals), the interdependent model of agency implies self-regulation behavior based on relatedness (e.g., maintain interpersonal harmony by adjusting goals to expectations of others).

One reason for cultural differences in self-regulation might be cultural variations in parenting (Trommsdorff et al., 2012). According to the theoretical framework of *the developmental niche* from Super and Harkness (1997), parenting is one of the factors which mediate the influence of culture on children's development. Keller et al. (2004) found in their study with samples of Cameroonian, Greek and Costa Rican mothers and infants cultural differences in parenting which were related to cultural differences in infants' self-regulation development. Cameroonian infants, who experience proximal parenting practices, developed self-regulation earlier than Greek infants, who experience distal parenting practices. Costa Rican infants, who experience a combination of distal and proximal parenting practices, lay between the Cameroonian and Greek groups.Relations between parenting and school achievement may also differ cross-culturally. Previous literature showed that restrictive control may have different effects on children's school achievement depending on the cultural context. In contrast to European and North-American contexts, restrictive control might be related to positive school achievement in Asian, African, or Latin American contexts (Dornbusch et al., 1987; Spera, 2005). However, studies investigating relations between restrictive control and developmental outcomes in Latin American contexts are still scarce. In their review on parenting studies in Chile, Bush and Peterson (2014) emphasize a need for cross-cultural research on parenting and child development based on adequate measurement of variables. Further, there are only few Latin American and even fewer Chilean studies on self-regulation and school achievement so far. Recently, studies with Mexican high school students discovered indirect relations of self-regulation on school achievement through resilience (e.g., Romero et al., 2013). Muñoz (2013) showed in a study with Chilean second graders positive relations between behavior regulation and school achievement. The present study investigated, whether maternal restrictive control is related to children's self-regulation and school achievement in Germany (a European context) and in Chile (a Latin American context) in similar or in different ways.

### Germany and chile as cultural contexts

Germany has been described as an independent sociocultural context, characterized by high independence and low interdependence values. For instance Hofstede (1980, 2001) ranked Germany as a country with high individualist values. In independent contexts, individualist values and a motivation for individual autonomy are typical (Trommsdorff, 2009). Parenting is directed to support the development of personal autonomy and self-reliance. Consequently, from infancy on, parents aim to foster autonomous self-regulation of their children, for instance by encouraging their children to sleep alone (Keller et al., 2011).

In contrast to Germany, Chile cannot be classified clearly as an independent or interdependent sociocultural context. In interdependent contexts, social orientations and a motivation for relatedness are typical (Trommsdorff, 2009). Hofstede (1980) characterized Chile as one of the most collectivistic countries. However, several more recent studies showed very high values of Chileans on both, independence and interdependence (Georgas et al., 2006; Kolstad and Horpestad, 2009; Schwinn, 2011). In countries undergoing rapid and extensive transformations, independent and interdependent values can combine (e.g., Trommsdorff and Kornadt, 2003). In Chile, political changes (the fall of the dictatorship and the re-democratization in 1990) in combination with the fast economic growth have led to a liberalization of social norms and to a rejection of authoritarian values (Martínez et al., 2006). This in turn is related to changes in parenting. It was found that today's Chilean parents report to be less authoritarian and to apply less power-assertive techniques than did their own parents (Martínez et al., 2006). Moreover, previous literature identified specific Latin American values, namely *simpatía* (respecting and sharing other's feelings)*, familismo* (strong family ties, commitment to the family), and *respeto* (avoidance of negative behaviors), which might underlie a motivation for interpersonal harmony in Chile (Triandis et al., 1984; Halgunseth et al., 2006). Thus, we could not be sure neither about the dominant psychological cultural values in Chile nor about their influence on cultural-specific parenting. Hence, the present study seeks to provide new insights by investigating relations between maternal restrictive control, self-regulation, and school achievement of Chilean children.

### Study aims and hypotheses

The present study aims to contribute to a better understanding of the role of self-regulation (i.e., behavior and emotion regulation) for children's school achievement as well as the role of maternal restrictive control for the development of self-regulation and school achievement in diverse cultural contexts. In this study, higher self-regulation was conceptualized as (a) higher behavior regulation, (b) lower usage of anger-oriented emotion regulation strategies, and (c) higher usage of problem-oriented emotion regulation strategies. In our cross-cultural analyses we focused on comparisons of mean values as well as on comparisons of relations between maternal restrictive control, self-regulation, and school achievement in a Chilean and a German sample.

First, concerning cross-cultural differences, clear hypotheses could not be formulated. As we stated above, there is not sufficient literature regarding cultural values in Chile available, so far. Hence, we formulated exploratory research questions. First, we analyzed if German and Chilean children differ in their self-regulation (research question 1). Second, we explored whether German and Chilean mothers differ in their restrictive control behavior toward their children (research question 2).

Second, in line with past research, we hypothesized that children's self-regulation is positively associated with their school achievement (hypothesis 1). Based on previous findings, we expected that the more restrictive control mothers prefer, the lower is their children's self-regulation (hypothesis 2). Furthermore, we hypothesized that the more restrictive control the mothers prefer, the lower is their children's school achievement (hypothesis 3). Moreover, we expected that the relations between mothers' restrictive control and children's school achievement are mediated by children's self-regulation (hypothesis 4).

Finally, we explored whether there are cultural differences in the relations between maternal restrictive control, children's self-regulation, and school achievement (research question 3).

## Methods

### Participants

The sample consisted of 76 German (31 boys, 45 girls) and 167 Chilean (56 boys, 111 girls) fourth graders, their mothers, and teachers. The mean age of the children was 10.21 years (*SD* = 0.44) in Germany and 10.16 years (*SD* = 0.42) in Chile. German children attended seven different fourth grade classes in four primary schools in a medium-sized town in Southern Germany. Chilean students attended nine different fourth grade classes in four primary schools (two public, two private) in a large city in Central Chile. The Chilean Sample was recruited in public and private schools to represent different socio-economic conditions of the Chilean educational system. To measure mother's level of education, ISCED-97 classification (Organization for Economic Co-operation Development., 1999) was used. In the German sample, five mothers (6.6%) had completed lower secondary level of education (= 2), ten (13.2%) upper secondary level (= 3), 23 (30.3%) post-secondary (= 4), and 38 (50%) had completed first stage of tertiary education (= 5). In Chile, three (1.8%) mothers had completed no school leaving certificate (= 0), 17 mothers (10.2 %) primary level of education (= 1), 49 (29.3%) lower secondary level of education (= 2), 48 (28.7%) upper secondary level of education (= 3), and 50 (29.9%) had completed first stage of tertiary education (= 5). The meaning of level of education is not simply comparable as variance and education system in the two cultural contexts differ considerably. Mothers and teachers of those children who participated in the study completed questionnaires for the assessment of maternal restrictive control, behavior regulation, and school achievement. The methods and procedures of this study were confirmed as ethically acceptable by the Ethics Committee of the University of Konstanz.

### Procedure

In Germany, the present study was part of a larger project which included for each child a group session at school which lasted about 1 h as well as a group session in rooms at the university lasting about 1.5 h. In Chile, children participated in group sessions at school which lasted about 1.5 h. In Germany and in Chile, group sessions included a nonverbal intelligence test and an emotion regulation questionnaire. Mothers and teachers answered paper-and-pencil questionnaires at home. Parents provided written informed consent prior to participation of their children and data was treated anonymously. Feedback of main results was provided to teachers and mothers who participated.

### Measures

#### Assessment of self-regulation

To assess behavior regulation, the *Strengths and Difficulties Questionnaire* (SDQ) from Goodman (1997) was administered. Teachers and mothers evaluated children's behavior regulation answering the h*yperactivity* s*cale* of the SDQ (five items on a 3-point scale from 1 = *not true* to 3 = *certainly true*; e.g., “Thinks things out before acting”). Scores of the hyperactivity scale were recoded such that a lower score of hyperactivity indicated a higher behavior regulation. Reliability analyses revealed a Cronbach's α of 0.83 for mothers' evaluation and a Cronbach's α of 0.76 for teachers' evaluation in the German sample. In the Chilean sample, for mothers Cronbach's α was 0.81 and for teachers Cronbach's α was 0.90. To increase the validity of the behavior regulation measure, mothers' and teachers' evaluations were used. This may take into account eventual variance in behavior regulation within individuals depending on context (i.e., home and school). Pearson correlations revealed that mothers' and teachers' evaluations of children's behavior regulation were significantly positively correlated in the German (*r* = 0.51, *p* < 0.01) as well as in the Chilean (*r* = 0.44, *p* < 0.01) sample. Accordingly, mothers' and teachers' evaluations of children's behavior regulation were averaged in each sample.

Children reported the use of emotion regulation strategies on the *Questionnaire for the Measurement of Stress and Coping in Children and Adolescents* (SSKJ 3-8) (Lohaus et al., 2006). Children were asked to imagine that they are in a stressful situation (problems with homework). Then they indicated how often (from 1 = *never* to 5 = *always*) they use anger-oriented strategies (six items; e.g. “I get mad and break something”) and problem-oriented strategies (six items; e.g., “I try to think of different ways to solve it”) to regulate their emotions. Reliability tests revealed satisfying results for anger-oriented strategies (Cronbach's α = 0.87 in the German sample; Cronbach's α = 0.73 in the Chilean sample) and for problem-oriented strategies (Cronbach's α = 0.80 in the German sample; Cronbach's α = 0.83 in the Chilean sample).

#### Assessment of school achievement

School achievement was assessed by language (German/Spanish) and mathematics grades. Grades were assessed by teachers' reports of the fourth grade midterm reports. In the German sample, grades were originally coded according to the German grade system ranging from 1 (= very good) to 6 (= not sufficient/fail). To facilitate the interpretation of the results, grades were recoded such that a higher score indicated higher school achievement. In the Chilean sample, grades were originally coded according to the Chilean grade system ranging from 1 (= not sufficient/fail) to 7 (= very good). To facilitate the comparability between the Chilean and the German sample, grades were z-standardized within both samples.

#### Assessment of maternal restrictive control

Maternal restrictive control was rated by mothers with the *Parenting Practice Questionnaire* (PPQ) by Robinson et al. (1995). Mothers answered items, indicating from 1 (= *never*) to 5 (= *always*), how often they show certain behaviors when interacting with their children. A scale with eight items was generated to assess maternal restrictive control (see Appendix A in Supplementary Material). Maternal restrictive control items implied direct parental control characterized by punishment and compliance without reasoning, e.g., “I use threats as punishment with little or no justification.” Reliability analyses revealed a Cronbach's α of 0.76 in the German sample and a Cronbach's α of 0.76 in the Chilean sample.

#### Assessment of intelligence

In order to assess nonverbal intelligence, the short version of the *CFT 20-R* (Weiß, 2006) was administered in the German sample. Weiß (2006) showed sufficient test-retest reliabilities of the CFT 20-R for German school children (*r* = 0.80−0.82). In the Chilean sample, the *Raven's Progressive Matrices* (Raven, 1957) were administered. Ivanovic et al. (2000) showed in a study with Chilean school children satisfactory test-retest reliability of the Raven Progressive Matrices (*r* = 0.45, *p* < 0.0001, for fourth graders). Nonverbal intelligence sum scores were z-standardized separately within the German and the Chilean samples, to facilitate comparability between samples.

#### Cultural equivalence of measures

To ensure comparability of the data from different cultures (i.e., Germany, Chile), the equivalence of instruments was maximized by a careful adaptation of instruments to the Chilean Sample. Furthermore, to test construct equivalence of instruments across the two cultural groups (Germany, Chile), factor congruence was evaluated by employing target rotations and computing Tucker's phi coefficients (van de Vijver and Leung, 1997; He and van de Vijver, 2012). Analyses of equivalence revealed a Tucker's phi coefficient of 1.00 for mothers' evaluation of children's behavior regulation and 1.00 for teachers' evaluation. Regarding emotion regulation, equivalence analyses revealed a Tucker's phi value of 0.98 for anger-oriented strategies and a Tucker's phi value of 0.95 for problem-solving strategies. The Tucker's phi value for maternal restrictive control was 0.97. Thus, in the present study the measures met the criteria of structural equivalence across cultures, as values above 0.95 are regarded as evidence for the similarity of factor structures (van de Vijver and Leung, 1997).

Analyses of covariance (ANCOVAs) were conducted to test cultural mean differences. Tests of cultural mean differences require scalar equivalence (He and van de Vijver, 2012). Therefore, scores were standardized with the ipsatization procedure to avoid cross-cultural differences due to response bias^1^ (van de Vijver and Leung, 1997; Fischer, 2004). For each individual means across all variables were computed and subtracted from each individual's raw score. Thus, the ipsatized score represents the person's position on this score in relation to the other variables. Furthermore, the resulting score was divided by each individual's standard deviation across all variables. Herewith, scores were adjusted for differences in the variation of answers around the mean (Fischer, 2004). As properties of ipsatized scores can distort statistical techniques involving correlations (Fischer, 2004), the ipsatized values were used for the ANCOVAs only. The relations between variables were tested with unstandardized original values.

### Data analysis

In order to test cultural mean differences in self-regulation (i.e., behavior and emotion regulation) and maternal restrictive control, ANCOVAs were conducted.

To test relations between maternal restrictive control, self-regulation (i.e., behavior and emotion regulation), and school achievement as well as to test if relations between maternal restrictive control and children's school achievement are mediated by self-regulation, mediation models were tested by using the bootstrapping method INDIRECT recommended by Preacher and Hayes (2008). The bootstrapping method has two strengths compared to conventional methods of mediation tests. First, multiple mediators can be tested in the same model at the same time. Second, bootstrapping avoids the assumption of a normal distribution of the indirect effects. Conventional methods often assume normal distributions. However, only in very large samples, sampling distributions can be expected to be normal distributed (Preacher and Hayes, 2008). Furthermore, PROCESS bootstrapping method by Hayes (2013) was used for moderator analyses to test whether relations were moderated by culture. Indirect effects, based on 95% confidence intervals (CI) derived from 5000 bootstrap samples, are significant when the CI values do not cross zero. Unstandardized coefficients (b) are reported for each regression equation.

## Results

### Cultural mean differences

To test cultural mean differences in self-regulation (i.e., behavior and emotion regulation) and maternal restrictive control (research questions 1 and 2), ANCOVAs with ipsatized values as well as with unstandardized original values were computed. All ANCOVAs included intelligence and age as covariates and gender as predictor variable. Means, standard deviations, and cultural mean differences of all variables under study are presented in Table 1. ANCOVAs with ipsatized values revealed that the behavior regulation of German children was rated significantly higher by mothers and teachers than the behavior regulation of Chilean children. Regarding cultural differences in anger-oriented emotion regulation, the ANCOVA revealed more anger-oriented emotion regulation strategies in German children in comparison to Chilean children. Regarding problem-oriented emotion regulation strategies, no significant effect for culture occurred. With respect to maternal restrictive control, ANCOVAs showed that Chilean mothers reported to use significantly more restrictive control than German mothers.

**Table 1:** **Means, standard deviations, and cultural mean differences**.

**Variable**	**Germany**		**Chile**		***F*(1, 237)**	***η*^2^**
	***M***	***SD***	***M***	***SD***		
Behavior	1.84	0.43	1.61	0.50	15.08^**^	0.06
regulation (M)	(2.43)	(0.48)	(2.17)	(0.55)	(14.25^**^)	(0.06)
Behavior	3.64	0.90	2.69	1.10	48.28^**^	0.17
regulation (T)	(2.70)	(0.41)	(2.23)	(0.61)	(38.50^**^)	(0.14)
Anger-oriented	1.46	0.60	1.32	0.48	4.23^*^	0.02
regulation	(1.95)	(0.10)	(1.89)	(0.07)	(0.23)	(0.00)
Problem-oriented	2.76	0.59	2.64	0.56	1.43	0.01
regulation	(3.67)	(0.11)	(3.87)	(0.08)	(5.29^*^)	(0.02)
Maternal restrictive	1.55	0.30	1.70	0.36	10.95^**^	0.04
control	(1.97)	(0.07)	(2.27)	(0.05)	(11.20^**^)	(0.05)

Ipsatized values; for reasons of clarity, a constant of 2.00 was added to all ipsatized values. Unstandardized original values are given in parentheses. N = 243, N (Germany) = 76, N (Chile) = 167; (M) = mothers' evaluations; (T) = teachers' evaluations;

* p < 0.05;

** *p < 0.01*.

Results of ANOCOVAs with unstandardized original values were consistent with the results of ANCOVAs with ipsatized values for children's behavior regulation and maternal restrictive control. ANOCOVAs with unstandardized original values showed no cultural mean differences for children's anger-oriented strategies and revealed more problem-oriented strategies in Chilean children in comparison to German children.

### Relations between restrictive control, self-regulation, and school achievement in cultural contexts

We tested hypotheses 1–4 by computing mediation models with maternal restrictive control as independent variable, school achievement (i.e., language and mathematics grades) as dependent variable, and self-regulation (i.e., behavior regulation, anger- and problem-oriented emotion regulation) as mediator variable. Intelligence, age, and gender were included as control variables. Mediation models were tested with the INDIRECT method, separately for the German and the Chilean samples and in each sample separately with language grade and mathematic grade as dependent variables.

**Figure 1:**
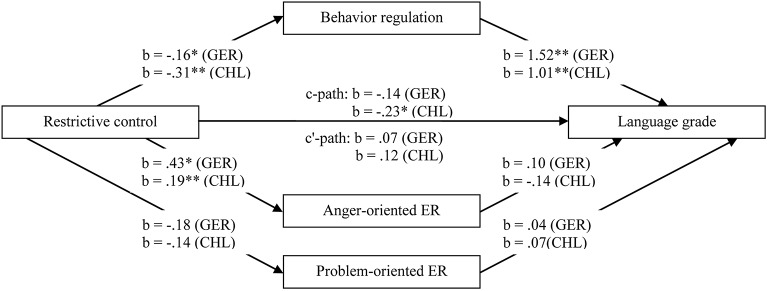
**Multiple mediation test of the relation between maternal restrictive control and language grade mediated by behavior regulation, anger- and problem-oriented emotion regulation**. Models were tested separately for the German and the Chilean samples. *N* (Germany) = 76; *N* (Chile) = 167; b = unstandardized regression coefficient, controlled for intelligence, age, and gender; GER = German sample; CHL = Chilean sample; ER = emotion regulation; ^*^p < 0.05; ^**^p < 0.01.

**Figure 2:**
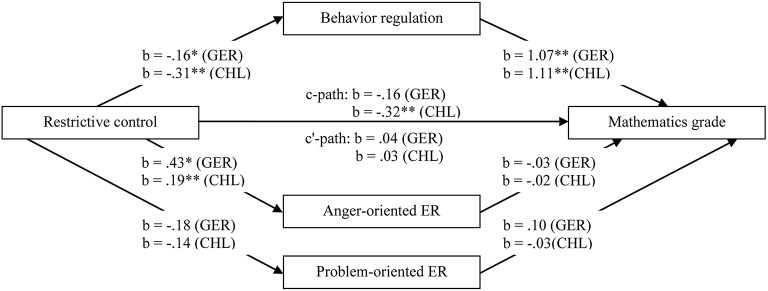
**Multiple mediation test of the relation between maternal restrictive control and mathematics grade mediated by behavior regulation, anger- and problem-oriented emotion regulation**. Models were tested separately for the German and the Chilean samples. *N* (Germany) = 76; *N* (Chile) = 167; b = unstandardized regression coefficient, controlled for intelligence, age, and gender; GER = German sample; CHL = Chilean sample; ER = emotion regulation; ^*^p < 0.05; ^**^p < 0.01.

The relations between maternal restrictive control, self-regulation, and school achievement are presented in Figure 1 (for language grades) and Figure 2 (for mathematics grades). In the German and in the Chilean sample, behavior regulation was significantly and positively related to language and mathematics grades. Emotion regulation strategies (i.e., anger- and problem-oriented emotion regulation) were not significantly related to language or mathematics grades, neither in Germany nor in Chile. In Germany as well as in Chile, we found negative relations between maternal restrictive control and behavior regulation and positive relations between restrictive control and anger-oriented emotion regulation. No significant relations between maternal restrictive control and problem-oriented emotion regulation occurred, neither in Germany nor in Chile. In Germany, restrictive control was not significantly associated with grades in language or mathematics. In Chile, maternal restrictive control was significantly and negatively related to language and mathematics grades.

In the German sample, significant indirect and negative effects of behavior regulation on the relations between restrictive control and school achievement (i.e., language and mathematics grades) occurred. Although neither the total effects *c*, nor the direct effects *c'* were significant, behavior regulation indirectly effected the relations between restrictive control and school achievement (language grade: indirect effect = −0.24, *SE* = 0.143, 95% CI [−0.60, −0.02]; mathematics grade: indirect effect = −0.17, *SE* = 0.109, 95% CI [−0.45, −0.01]). The models accounted for 35% of the variance (Adjusted *R*^2^ = 0.35) of children's mathematics grade and for 31% of the variance (Adjusted *R*^2^ = 0.31) of children's language grade in the German sample.

In the Chilean sample, behavior regulation significantly mediated the relations between maternal restrictive control and school achievement (i.e., language and mathematics grades). The total effects *c* were significant, while the direct effects *c'* were non-significant. Thus, behavior regulation was a significant mediator (language grade: indirect effect = −0.311, *SE* = 0.07, 95% CI [−0.47, −0.18]; mathematics grade: indirect effect = −0.35, *SE* = 0.07, 95% CI [−0.51, −0.22]). The models accounted in the Chilean sample for 44% of the variance (Adjusted *R*^2^ = 0.44) of children's mathematics grade and for 38% of the variance (Adjusted *R*^2^ = 0.38) of children's language grade.

To test whether the relations were moderated by culture (research question 3) moderated mediation models were conducted with the whole sample with the PROCESS method. Here, maternal restrictive control was included as independent variable, school achievement (i.e., language and mathematics grades) as dependent variable, self-regulation (i.e., behavior regulation, anger- and problem-oriented emotion regulation) as mediator variable, and culture (i.e., Germany, Chile) as moderator variable. Intelligence, age, and gender were included as control variables. Results of the moderated mediation models revealed no significant moderation of culture on the relations of the model (see Appendix B in Supplementary Material). Thus, relations between variables did not differ significantly between the German and the Chilean sample.

## Discussion

The present study revealed that behavior regulation and anger-oriented emotion regulation were higher for German children than for Chilean children. Chilean mothers used more restrictive control than German mothers. In both cultural contexts, children's behavior regulation and school achievement were related positively. Maternal restrictive control was related negatively to children's behavior regulation and positively to anger-oriented emotion regulation. Indirect negative effects of behavior regulation on relations between restrictive control and school achievement were found. Overall, the study confirmed the hypotheses that maternal restrictive control was related negatively to children's self-regulation and that behavior regulation was related positively to school achievement. Regarding our research questions on cross-cultural differences, we found cultural mean differences but no cultural differences in the relations among the variables.

One reason for the higher behavior regulation of German children might be their socialization in an independence-oriented context. As the development of personal autonomy is central for socialization in independence-oriented contexts, German parents aim to support the development of behavior regulation from an early age on (e.g., Keller et al., 2011). Due to lacking literature, no clear conclusions about socialization conditions in Chile can be drawn. As there might exist independence along with interdependence values in Chile (Georgas et al., 2006; Kolstad and Horpestad, 2009; Schwinn, 2011), both might influence behavior regulation in different ways or might even be contradictory. This might be a reason for the lower behavior regulation in Chilean children.

The higher usage of anger-oriented strategies in German children in comparison to Chilean children is in line with the notion that the expression of frustration and anger differs depending on the respective cultural values of interdependence and independence (Cole et al., 2006; Trommsdorff, 2009, 2012; Trommsdorff and Cole, 2011). The cultural model of independence allows for the expression of anger and frustration as this can be instrumental to assert individual goals. In contrast, the cultural model of interdependence reinforces an endorsement of interpersonal harmony and discourages the expression of anger (Trommsdorff, 2009, 2012). Thus, German children might use anger-oriented strategies more often than children from interdependent contexts because of their socialization experiences encouraging self-assertion. For instance, previous studies comparing German and Japanese or German and Indian preschool children also showed higher anger expression of German children (see Trommsdorff, 2009, 2012; Trommsdorff and Cole, 2011). In Chile, the development of anger-oriented emotion regulation might be influenced by values of interdependence. Moreover, Latin American specific values as *simpatía* and *respeto* might play an additional role in striving for interpersonal harmony and avoiding negative emotional expressions as anger (Triandis et al., 1984; Halgunseth et al., 2006). This might be another reason why Chilean children used less anger-oriented strategies than German children in the present study. We are aware of the difficulty to interpret cultural mean comparisons for children's anger-oriented and problem-oriented emotion regulation strategies as scalar equivalence is not ensured for these scales and there might exist an acquiescence bias. This means that the higher anger-oriented emotion regulation of German children in comparison to Chilean children might be (in part) a result of cultural differences in acquiescence. Further, there might exist cultural differences in problem-oriented emotion regulation strategies which may not be found due to acquiescence bias.

Chilean mothers used more restrictive control than German mothers. This finding confirms previous studies which found Latino parents to use more restrictive control than European-American parents (for a review see Halgunseth et al., 2006). Recent literature argued that political and economic changes in Chile have led to a decline of parental restrictive control (Martínez et al., 2006). Based on our results it seems that even if today's Chilean mothers use less restrictive control than their own mothers (Martínez et al., 2006), they still use more restrictive control than German mothers do.

As hypothesized, behavior regulation was positively associated with school achievement (i.e., language and mathematics grades) in both samples. This result underlines the central function of behavior regulation for academic competences. However, contrary to our hypotheses, no relations between emotion regulation strategies and school achievement occurred, neither in Germany nor in Chile. This finding brings up the question whether behavior regulation is more relevant for school achievement than emotion regulation. Future studies should investigate whether emotion regulation effects school achievement indirectly via behavior regulation (McClelland and Cameron, 2011) or via social competences (Eisenberg et al., 2005) and also should consider the high context specificity of the relation between emotion regulation and school adjustment (Hernández et al., 2016).

Furthermore, in line with our hypotheses, maternal restrictive control was related negatively to behavior regulation and positively to anger-oriented emotion regulation, both in Germany and in Chile. These results fit with the theoretical assumption that maternal restrictive control may undermine children's internalization of adequate self-regulation processes.

As hypothesized, we found negative relations between maternal restrictive control and school achievement (i.e., language and mathematics grades) in Chile. However, these relations were not found for the German sample. This result is in contrast to previous assumptions (e.g., Dornbusch et al., 1987) about positive relations between parental restrictive control and school achievement in Latinos. The present study revealed that maternal restrictive control was associated negatively with children's self-regulation as well as with school achievement in a Latin American context (i.e., Chile).

To conclude, the present study revealed cross-cultural differences as well as cross-cultural similarities. Cross-cultural mean differences occurred in maternal restrictive control and children's self-regulation (i.e., behavior regulation, anger-oriented emotion regulation). Further, relations between maternal restrictive control, children's self-regulation, and school achievement did not differ between cultures. The similarity of the relations was shown by moderated mediation models which revealed no significant interactions of culture. Thus, the present study underlines the importance to distinguish among level-oriented analyses of cultural mean differences and structure-oriented analyses of cross-cultural similarities and differences in relations among variables (van de Vijver, 2009). In this study, although level-oriented analyses showed cultural mean differences, structure-oriented analyses revealed no cultural differences. Both types of analyses are necessary and complement each other.

### Strengths and limitations

The negative relations between maternal restrictive control and children's self-regulation in diverse cultural contexts could be bidirectional. That is, maternal restrictive control might induce lower behavior regulation in children; however children's behavior regulation may also influence maternal restrictive control. Previous literature argued that parents' restrictive control might be a consequence of children's low behavior regulation (Karreman et al., 2006). Further, the cultural meaning (and value) of restrictive control should be ascertained in future studies. Moreover, there might be cross-cultural differences in the bidirectionality of parent-child relations (Trommsdorff and Kornadt, 2003). Thus, future cross-cultural research, using longitudinal designs and observational measures, is needed to distinguish parenting effects from children's characteristics regarding maternal restrictive control and self-regulation.

Furthermore, teacher's evaluation of children's school achievement could influence their rating of children's behavior regulation. Moreover, school system and scholastic learning could influence self-regulation. Therefore, mothers', teachers', and children's evaluation of children's self-regulation (i.e., behavior and emotion regulation) were included. The study measured children's self-regulation by using multiple sources (children, mothers, and teachers) in order to take into account eventual variance in self-regulation within individuals depending on different contexts (i.e., home and school). Future studies should include direct as well as multiple-methods strategies to assess behavior and emotion regulation.

Moreover, the different sample sizes of the German and Chilean samples could account for differences in the magnitudes of effects between the samples. However, moderation analyses showed no significant moderation of culture on the relations of the model. Thus, relations between variables did not differ significantly between the German and the Chilean sample. Nevertheless, magnitudes of indirect effects might be higher in Chile due to the larger sample size.

## Conclusions

The findings of the present study indicate that restrictive control and behavior regulation might play a crucial role for school achievement in a European as well as in a Latin American context. Thus, an important practical implication of the present study is that interventions to strengthen children's behavior regulation may be an effective way to promote children's school achievement. Hence, school curricula designed to improve children's behavior regulation (Blair and Razza, 2007) as well as intervention programs which have been shown to improve behavior regulation in school-age children (e.g., Diamond et al., 2007) might help children to succeed in school. Moreover, the results show how relevant parenting and culture are for children's self-regulation and their school achievement. Thus, intervention programs should be adapted to individuals' cultural background and should include children as well as parents and teachers.

## Author contributions

MW: Principal investigator of the research project in Chile, development of study design and selection of instruments for the study in Chile, literature review, conceptualization of research question, responsible for data collection of the Chilean sample, data analysis and interpretation, preparation of written manuscript. GT: Principal investigator of the research project in Germany, development of study design and selection of instruments for the German study, conceptual input, general supervisory input, review of manuscript. LM: Assistance in the organization of the research project in Chile, assistance in adaptation of instruments to the Chilean sample, assistance in data collection of the Chilean sample, review of manuscript.

### Conflict of interest statement

The authors declare that the research was conducted in the absence of any commercial or financial relationships that could be construed as a potential conflict of interest.

## Footnotes

^1^ANCOVAs with ipsatized values as well as ANOCOVAs with unstandardized original values were conducted. When results of ANCOVAs with ipsatized values and unstandardized values are consistent, absence of acquiescence bias is ensured. When results of ipsatized values and unstandardized values differ, there might exist an acquiescence bias.
