# Supplementary material for: Children's Self-Regulation and School Achievement in Cultural Contexts: The Role of Maternal Restrictive Control
Source: Front Psychol. 2016 May 31;7:722. doi: 10.3389/fpsyg.2016.00722 (PMC4885849; doi:10.3389/fpsyg.2016.00722)
Supplement: Supplementary file 1 [file DataSheet1.docx]

Appendix A

*Items of the Restrictive Control Scale*

| 1. I guide my child by punishment more than by reason. |
| --- |
| 1. I punish by taking privileges away from my child with little if any explanation. |
| 1. I yell or shout when my child misbehaves. |
| 1. I scold and criticize to make my child improve. |
| 1. I punish by putting my child off somewhere alone with little if any explanation. |
| 1. I scold or criticize when my child's behavior doesn't meet my expectations. |
| 1. I use threats as punishment with little or no justification. |
| 1. When my child asks why he/she has to conform, I state: because I said so, or I am your parent and I want you to. |

*Note.* This *Maternal Restrictive Control Scale* with eight items was generated for the present study. Items were derived from the *Parenting Practice Questionnaire* (PPQ) from Robinson et al. (1995). Mothers answered the items on a 5-point rating scale (from 1 = *never* to 5 = *always*).

Appendix B

*Interaction effects of moderated mediation models with culture as moderator variable*

|  | Behavior regulation | | Anger-oriented ER | | Problem-oriented ER | | Language grade | | Mathematics grade | |
| --- | --- | --- | --- | --- | --- | --- | --- | --- | --- | --- |
|  | b | 95% CI | b | 95% CI | b | 95% CI | b | 95% CI | b | 95% CI |
| Restrictive control × culture interaction | .15 | -.05, .35 | .23 | -.17, .62 | -.06 | -.51, .39 | -.06 | -.48, .37 | .01 | -.37, .39 |
| Behavior regulation × culture interaction | − | − | − | − | − | − | .24 | -.33, .81 | -.16 | -.67, .36 |
| Anger-oriented ER × culture interaction | − | − | − | − | − | − | .23 | -.05, .50 | -.01 | -.26, .24 |
| Problem-oriented ER × culture interaction | − | − | − | − | − | − | -.01 | -.29, .26 | .15 | -.10, .40 |

*Note. N* = 243, *N* (Germany) = 76; *N* (Chile) = 167; ER = emotion regulation; b = unstandardized regression coefficient, controlled for intelligence, age, and gender.
